# The Role of Nitrogen Dopants in ZnO Nanoparticle-Based Light Emitting Diodes

**DOI:** 10.3390/nano12030358

**Published:** 2022-01-22

**Authors:** Islam Mohammad Shafiqul, Raj Deep, Jie Lin, Toshiyuki Yoshida, Yasuhisa Fujita

**Affiliations:** 1 Interdisciplinary Graduate School of Science and Engineering, Shimane University, 1060 Nishikawatsu, Matsue 690-8504, Japan; 2 Graduate School of Natural Science and Technology, Shimane University, 1060 Nishikawatsu, Matsue 690-8504, Japan; n21d103@matsu.shimane-u.ac.jp (R.D.); yosisi@riko.shimane-u.ac.jp (T.Y.); 3 S-Nanotech Co-Creation Co., Ltd., 1060 Nishikawatsu, Matsue 690-0823, Japan; linjie3000@gmail.com

**Keywords:** ZnO nanoparticles, nitrogen doping, electroluminescence, photoluminescence, light-emitting diodes

## Abstract

In this work, nitrogen-doped ZnO nanoparticles were synthesized in various conditions by the gas evaporation method with DC arc plasma. Nitrogen concentrations of 6.38 × 10^18^ cm^−3^ to 2.6 × 10^19^ cm^−3^ were obtained at a chamber pressure of 150 torr, using arc currents of 20 A to 70 A. The intensities of local vibrational modes at 275 cm^−1^ and 581 cm^−1^ in the Raman spectra of ZnO nanoparticles showed a dependency on the nitrogen concentration in the ZnO nanoparticles. The ratios of donor–acceptor pair and exciton emissions in the photoluminescence spectra of nitrogen-doped ZnO nanoparticles, and the electroluminescence of light-emitting diodes based on these nanoparticles, were nearly proportional to the Raman peak’s intensity at 275 cm^−1^. The results indicated that the nitrogen dopants in the ZnO nanoparticles were acting as an acceptor.

## 1. Introduction

ZnO is currently of great interest for the development of novel solid-state lighting devices. ZnO has a wide bandgap of 3.37 eV and a stable exciton binding energy of 60 meV for light emission in the near-UV spectral range at room temperature [1,2]. One hurdle in ZnO light device development is the difficulty in fabricating p-type ZnO. This difficulty limits the application of ZnO in common optical devices, such as LEDs, that require precise doping. To date, protocols that achieve reproducible and stable p-type ZnO have not been developed. One reason for the slow development is due to the defects in ZnO, such as oxygen vacancy (V_O_), zinc interstitial (Zn_i_), etc., and that there are few candidates for shallow acceptors [3]. The most reliable dopants for p-type ZnO are group V elements, such as phosphorus (P) [4], arsenic (As) [5], antimony (Sb) [6], and nitrogen (N) [7]. Nitrogen is the most suitable p-type dopant due to its atomic size being similar to that of oxygen. The behavior of nitrogen dopants in ZnO has been discussed by theoretical calculations, such as the ab initio electronic bandstructure method [8] and density functional theory [9]. Incorporating nitrogen dopants and co-dopants into ZnO has previously been reported [10,11,12]. Some reports have shown nitrogen-doped p-type ZnO- or ZnMgO-based LEDs with single-crystal films using epitaxial growth technologies [13,14,15]. Unfortunately, single-crystal substrates and epitaxial growth technologies require strick fabrication controls and are currently not cost-effective. On the other hand, the fabrication of scalable LEDs using nanoparticles (NPs) is inexpensive and can be fabricated in atmospheric conditions.

Currently, nitrogen-doped ZnO NPs are a unique and attractive issue. Various fabrication methods have been developed for synthesizing nitrogen-doped ZnO NPs, such as radio frequency (RF) thermal plasma, hydrothermal-ammonolysis, and Nd:YAG laser ablation [16,17,18]. Our group has successfully developed nitrogen-doped ZnO NPs using a DC arc plasma gas evaporation method. Additionally, we have developed several protocols for the fabrication of ZnO NPs with both p-type and n-type conductivity [19,20,21]. Though it contained the influence of many boundaries, Itohara et al., reported the p-type conductivity (mobility 5~7 cm^2^/Vs and carrier concentration + 2.0 × 10^12^ ~ + 2.7 × 10^12^ cm^−3^) of nitrogen-doped ZnO NPs films [22]. We previously demonstrated the operation of semiconductor devices, such as thin-film transistors [22] and UV LED [23], using nitrogen-doped ZnO NPs for p-type layers. Fujita et al., demonstrated near-ultraviolet, homojunction LEDs based on nitrogen-doped ZnO NP layers on a GZO (Ga-doped ZnO) film by a simple coating process [23]. Shafiqul et al., demonstrated all nanoparticle-based homojunction UV LEDs using Ga-doped ZnO NPs (n-type) and nitrogen-doped ZnO NPs (p-type) [24]. However, due to the difficulty of measuring a single nanoparticle’s electrical properties, such as carrier concentration and mobility, the role of nitrogen in p-type characteristics of ZnO NPs is not yet understood.

In this study, to better understand the role of nitrogen in ZnO NPs, we synthesized nitrogen-doped ZnO NPs in various conditions using the gas evaporation method. We investigated the material property dependencies of the ZnO NPs and ZnO NP-based LEDs on nitrogen concentrations of ZnO NPs.

## 2. Materials and Methods

To fabricate nitrogen-doped ZnO NPs, we used an arc vapor deposition method (ULVAC Inc., Model No-GE-970, Chigasaki, kanagawa, Japan). We previously detailed the arc vapor method process and mechanism for ZnO NPs synthesis [19,20]. Briefly, zinc metal (Nilaco Corporation, Chuo-Ku, Tokyo, Japan. Zn-99.99%) was used for the Zn source, and dry air was used for the oxygen and nitrogen sources. The dry air was allowed to flow into the chamber to control the dopants during the arc reaction. A carbon cathode was placed above the Zn source in the chamber, and a potential difference was applied between the carbon cathode and Zn source to create a current arc. The plasma generated by the current arc caused a reaction with the Zn and dry air. In the reaction, O_2_ and N_2_ radicals were generated from the dry air by the arc plasma and incorporated into nanoparticulate crystalline Zn from the surface of the Zn source [19]. The resulting reaction produced ZnO NPs of various sizes, which incorporated nitrogen atoms. In our investigation, we created nitrogen-doped ZnO NPs with different nitrogen and oxygen concentrations by controlling the arc current and chamber pressure. For generating different samples, arc currents were varied between 20 A and 70 A. The chamber pressure was regulated between 75 and 760 torr using a rotary pump and control valve. For all NP synthesis conditions, the airflow rate inside the chamber was held at a constant 5 L min^−1^.

**Figure 1:**
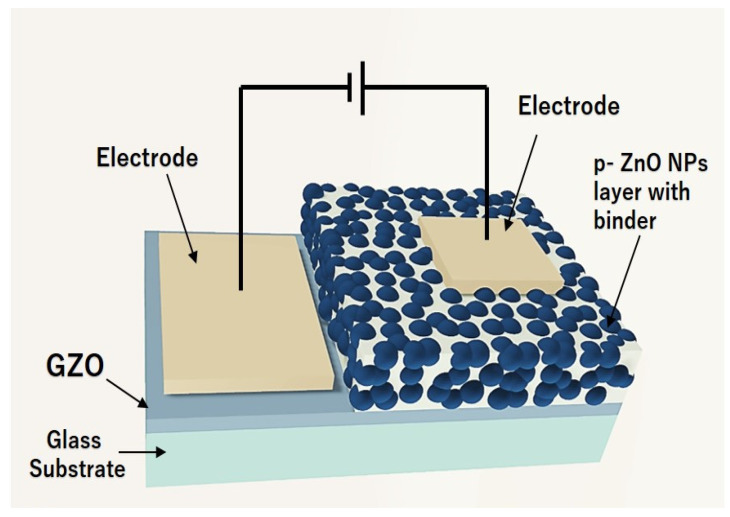
Schematic view of ZnO NP LEDs.

We used the ZnO NPs to fabricate LED devices to evaluate the p-type characteristics of nitrogen-doped ZnO NPs. An illustration of the device build is shown in Figure 1. To create the LEDs, we first prepared a dispersion by mixing isopropyl alcohol (IPA) (0.3 mL), and binder (0.1 g) (Silsesquioxane OX-SQ SI 20; Toagosei Co., Ltd., Minato-ku, Tokyo, Japan) with nitrogen-doped ZnO NPs (0.05 g). The ZnO NP dispersions were coated on the GZO electrode films (thickness of 500 nm) using the spin coating. The GZO films were prepared using a 5% Ga-doped ZnO target on a 500 µm-thick glass substrate by RF magnetron sputtering (Canon Anelva Corporation, Kawasaki, Kanagawa, Japan. Model-400S) at a temperature of 300 °C. The spin coating process followed a two-step rotational condition at an initial speed of 1000 rpm for 5 s, which was accelerated to a final speed of 4000 rpm for 10 s. The nitrogen-doped ZnO NP-coated layers were sintered by a hot plate at ~300 °C. Gold (Au) contact electrodes of 30 nm thickness were deposited on both the p-type layer and the GZO film using a vacuum deposition procedure. The previous research articles examined the ohmic behavior between the contact electrode (Au) and the p-ZnO NP film [22].

We measured the nitrogen concentration and characterized the corresponding material properties of the nitrogen-doped ZnO NPs. Nitrogen concentration in ZnO NP powders were measured by a thermal conductivity detector (EMGA-830 O/N analyzer, Horiba, Minami-ku, Kyoko, Japan). Raman spectra of the NPs were also measured using the 532 nm laser line of a high-resolution Raman confocal system (Nanofinder 30, Tokyo Instruments, Edogawa-ku. Tokyo, Japan). Photoluminescence (PL) spectra of the NPs were acquired at an excitation wavelength of 325 nm using a spectrofluorometer (FluoroMax-4, Horiba, Minami-ku, Kyoto, Japan. A diffractometer (SmartLab, Rigaku, Akishima, Tokyo, Japan) with Cuk_α_ radiation was used for measuring X-ray diffraction (XRD) in the ZnO NP powder samples. The size and shape of NPs were observed using a field emission scanning electron microscope (FESEM; JSM-7001FA, 5 KV, JEOL, Akishima, Tokyo, Japan). We measured the current–voltage (*I*–*V*) characteristics of the fabricated LEDs using a parameter analyzer (B2900A High-Resolution SMU, Keysight Technologies, Hachioji, Tokyo, Japan). Finally, we evaluated the electroluminescence (EL) spectra of the LEDs fabricated from the ZnO NPs. The EL spectra were measured from the top side of the p-contact electrode at room temperature using a spectrometer (QE65000, Ocean Optics, Ontario, NY, USA). The EL power of the LEDs was measured from the bottom side through a glass substrate using a Si photodiode (S2281, Hamamatsu Photonics, Hamamatsu, Shizuoka, Japan).

## 3. Results and Discussion

**Figure 2:**
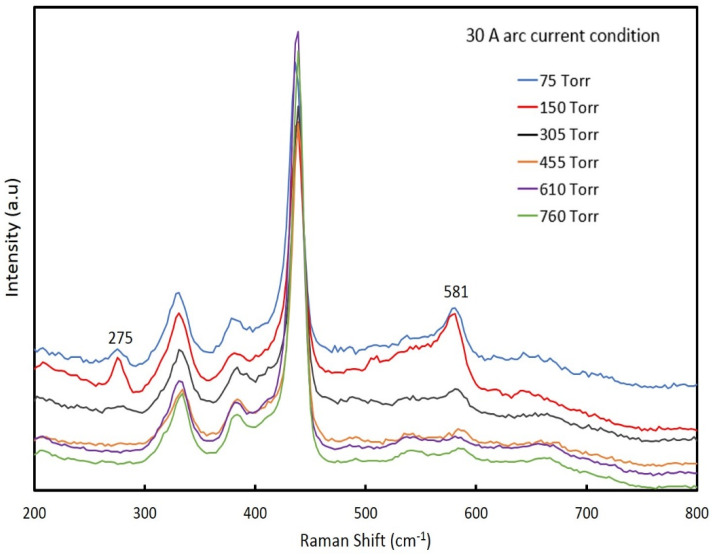
The Raman spectra of ZnO NPs prepared at an arc current of 30 A and chamber pressures between 75 to 760 torr.

Figure 2 shows the Raman spectra of ZnO NPs synthesized at an arc current of 30 A with different chamber pressures ranging from 75 torr to 760 torr. The Raman active, non-polar phonon modes E_2_ (high) were observed at 438 cm^−1^, and the polar A_1_ (TO) and E_1_ (LO) optical modes appeared at 380 cm^−1^ and 584 cm^−1^, respectively [25]. The second-order scattering feature at 332 cm^−1^ was attributed to the multi-phonon scattering process [26]. In addition, the expected strongest nitrogen-related local vibrational modes (LVMs) in the Raman spectra were located at 275 cm^−1^ and 581 cm^−1^ [27]. In this case, the nitrogen content in the ZnO NPs at a chamber pressure of 150 torr was higher than those of the other chamber pressures. Presumably, this was related to the lifetimes of N_2_ radicals being extended at the lower chamber pressure through fewer collisions, which would increase nitrogen incorporation. At 75 torr, the reaction time was shorter due to a higher gas flow rate, leading to lower nitrogen content than at a pressure of 150 torr. If nitrogen acted as an acceptor, these results were consistent with the results of Itohara et al., where the sample fabricated at 150 torr exhibited p-type conduction, while the sample fabricated at 610 torr exhibited n-type conduction [22].

**Figure 3:**
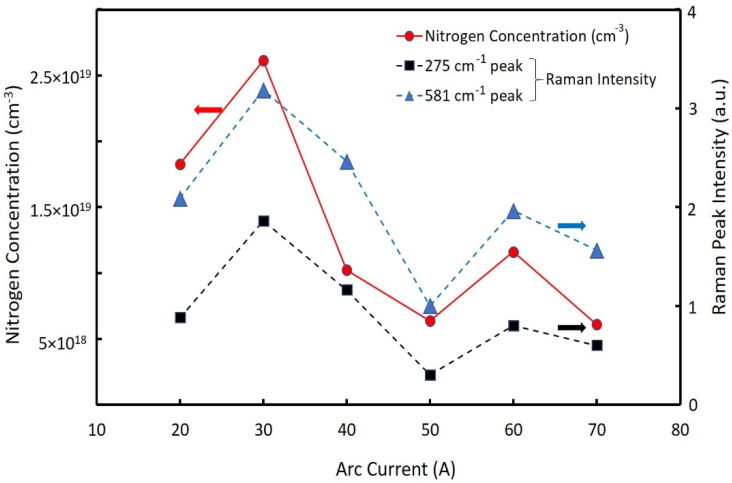
Nitrogen concentrations (solid line) and Raman peak intensities (275 cm^−1^ and 582 cm^−1^) (dotted lines) as a function of the arc current (20~70 A).

We investigated the relationship between arc current and nitrogen content of the NPs. Different batches of nitrogen-doped ZnO NPs were created at arc currents between 20 A to 70 A. The chamber pressure for all samples was held constant at 150 torr. Figure 3 shows the relationship between the nitrogen concentration and the nitrogen-related LVM Raman peaks (275 cm^−1^ and 581 cm^−1^) of the NPs fabricated at the different arc currents. The nitrogen concentrations ranged from 6.38 × 10^18^ cm^−3^ to 2.6 × 10^19^ cm^−3^. The LVM peak intensities of the Raman spectra depended on the nitrogen concentration in the ZnO NPs. The maximum nitrogen concentration was observed at an arc current of 30 A. Thus, the intensity values for the LVM peak (275 cm^−1^ and 581 cm^−1^) correlated with the nitrogen concentration in the ZnO NPs. This was consistent with previous literature, which demonstrated that the nitrogen concentration had a linear dependence on the Raman spectra intensity of the LVM [27]. The nitrogen concentration at the arc current of 30 A was approximately 2.6 × 10^19^ cm^−3^, which was similar to the value of nitrogen concentration in nitrogen-doped ZnO NPs previously reported [20]. Note that the nitrogen concentration, measured by the thermal conductivity method, contained surface-absorbed species of nitrogen molecules. The 275 cm^−1^ peaks may have originated from nitrogen substitution of oxygen, whereas the broad Raman peak at 581 cm^−1^ was related to the defect complexes [28]. Therefore, we used the intensities of Raman peaks at 275 cm^−1^ as the relative value of the nitrogen concentrations that doped into the ZnO NPs for the following discussions.

**Figure 4:**
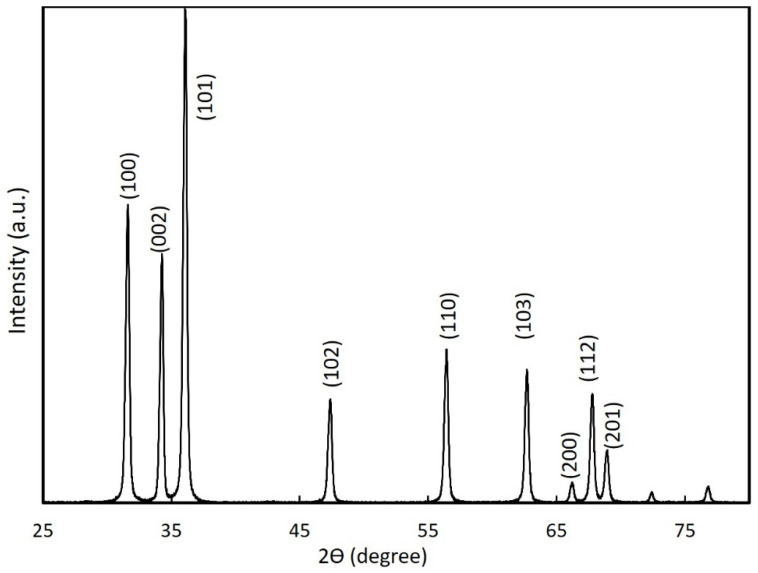
XRD of nitrogen-doped ZnO NPs synthesized at a chamber pressure of 150 torr and an arc current of 30 A.

We analyzed the structural morphology of the nitrogen-doped ZnO NPs synthesized at a pressure of 150 T and arc current of 30 A using XRD, as shown in Figure 4. The XRD peaks for the powder ZnO NP samples showed the hexagonal wurtzite crystal structure with proper orientation, which was consistent with the standard JCPDS data (36-451) [29] for ZnO. No additional peaks were present due to other impurities. The XRD diffraction peak (002) of nitrogen-doped ZnO NPs was centered at approximately 34.31°, which was shifted compared to the known diffraction peak (002) for bulk ZnO (34.42°) [30]. This shift was evidence of the incorporation of nitrogen into the ZnO NP crystal lattice and was consistent with Raman spectroscopy results.

**Figure 5:**
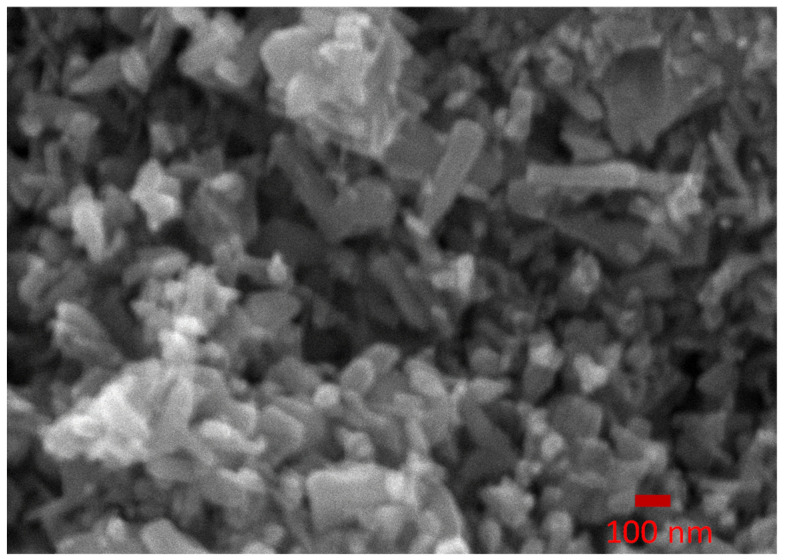
The SEM image of ZnO nanoparticles synthesized at a chamber pressure of 150 torr and arc current of 50 A.

An SEM image of ZnO nanoparticles synthesized at a chamber pressure of 150 torr and arc current of 50 A is shown in Figure 5. The SEM image shows that the ZnO NPs have different morphologies, such as rods and spheres. The average size of the ZnO NPs was around 100–200 nm.

**Figure 6:**
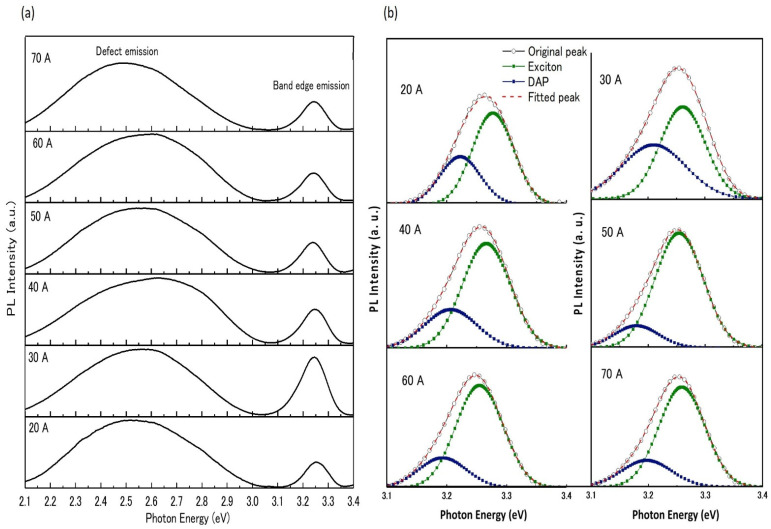
Room-temperature PL spectra of ZnO NPs prepared at a chamber pressure of 150 torr and with arc currents of 20 A to70 A. (**a**) The full spectra as measured by the spectrometer; (**b**) the Gaussian band deconvolution for NBE emission.

Figure 6a shows the room temperature PL spectra for nitrogen-doped ZnO NPs generated at a chamber pressure of 150 torr and arc currents between 20 A and 70 A. The deep level emission was caused by defect-related transitions in the lattice or near the surface of the ZnO, such as oxygen vacancies (V_O_), zinc vacancies (V_Zn_), and zinc interstitials (Zn_i_) [31]. The near-band-edge (NBE) emission peak shifted toward the lower energy side due to its appearance at the donor–acceptor pair (DAP) transition [32]. The NBE emission of ZnO NPs was found to be strongest at an arc current of 30 A, compared to other currents. 

The acceptor nitrogen incorporation in the ZnO NPs crystals was revealed in their deconvolution of photoluminescence (PL) spectra at (NBE) emission through the appearance of DAP recombination [33,34,35]. Figure 6b represents the NBE emission and the Gaussian deconvolution peaks corresponding to the exciton emission and DAP emission. The intensity of the DAP emission increased with the addition of nitrogen in the ZnO NP lattice, which was reflected in the acceptor levels of ZnO NPs. Zeuner et al., suggested the nature of the DAP band at 3.24 eV for nitrogen-doped ZnO [36]. We observed that the DAP band was centered at approximately 3.23 eV. The strong 3.23 eV emission intensity confirmed the involvement of nitrogen acceptors [37].

**Figure 7:**
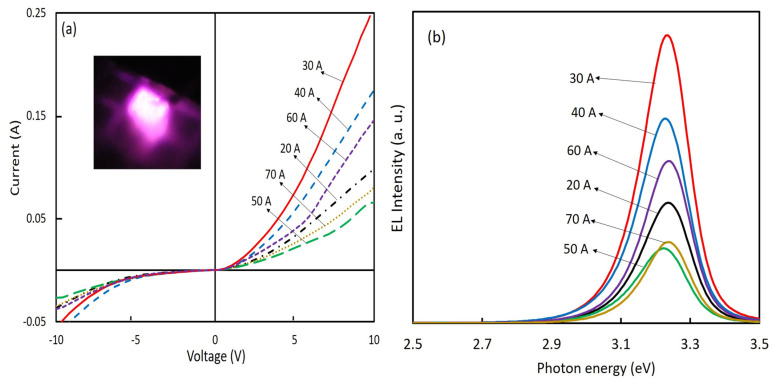
(**a**) *I–V* characteristics of the ZnO NP-based LEDs (inset is the photo of LED emission); (**b**) EL spectra of ZnO NPs LEDs using nitrogen-doped ZnO NPs prepared at a chamber pressure of 150 torr and with arc currents ranging from 20 A to 70 A.

We fabricated LEDs using nitrogen-doped ZnO NPs. The ZnO NPs acted as hole injection layers. Therefore, we chose to use NPs fabricated at a chamber pressure of 150 torr and arc currents of 20 A to 70 A, which had the highest nitrogen concentrations. Figure 7a displays the *I–V* properties of the fabricated ZnO NP-based LEDs. Figure 7b shows the corresponding EL spectra of the LEDs at a forward bias voltage of 10 V. The *I–V* characteristics demonstrated a diode rectification character at room temperature and under dark conditions. The device resistivities under forwarding bias were near proportional to the EL intensities. The leakage currents were observed from the devices, but the meaning of the leakage current variety for various conditions of ZnO NPs was still unknown and requires further investigations. The EL spectra showed only the near-band-edge UV emission, and no deep-level emissions in the PL spectra (excited by a weak power source) were observed. It was considered that the deep-level transition for EL emissions was saturated by the higher injected carrier density [23]. The EL peak energy of LEDs shifted toward the lower energy side compared to the PL spectra, due to the heating effect of temperature rises during operation of the devices [15,24].

**Figure 8:**
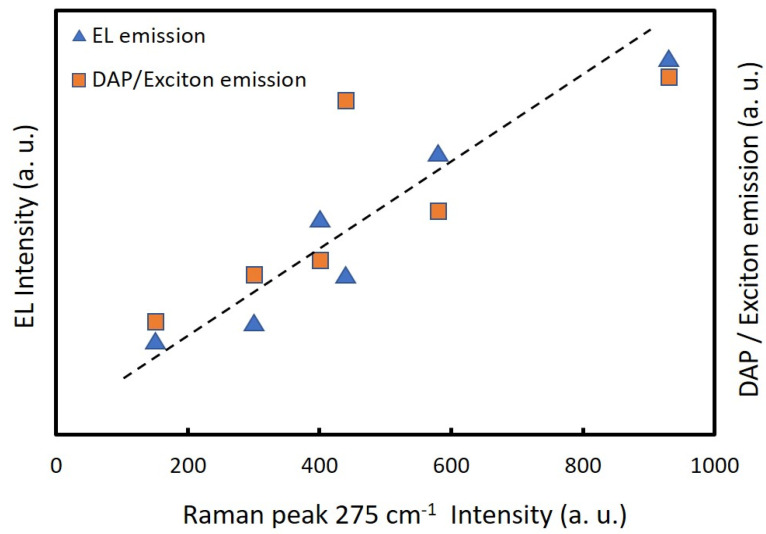
Ratios of DAP/exciton emission calculated from Figure 6b and EL intensities of the LEDs versus 275 cm^−1^ Raman peak intensities of the nitrogen-doped ZnO NPs prepared at a chamber pressure of 150 torr and the arc currents of 20 A to 70 A.

Figure 8 shows the dependence of EL intensities on the nitrogen concentrations in ZnO NPs (275 cm^−1^ Raman peaks) and the ratiometric of the DAP emission and exciton emission, calculated from Figure 6b, versus the 275 cm^−1^ Raman peak intensity of the respective sample. The ratios of DAP/exciton emission intensity were near proportional to the nitrogen concentrations of the ZnO NPs (275 cm^−1^ peak intensities of Raman spectra). This suggested that the nitrogen concentrations of the ZnO NPs correlated with the donor or acceptor concentrations. Thus, the results indicated that the incorporated nitrogen acted as an acceptor in the ZnO lattice. This also clearly showed that the EL intensities of the LEDs were proportional to the nitrogen concentrations of the ZnO NPs. 

As mentioned above, the densities of nitrogen in ZnO NPs contributed to increasing acceptors, the conductivity of p-type layers, and the EL intensities of LEDs. Taking into account these results, the authors conclude that nitrogen dopant acts as an acceptor in ZnO NPs, and the nitrogen-doped ZnO NP layer functions as a hole injection layer in the LED devices.

## 4. Conclusions

Nitrogen-doped ZnO nanoparticles were successfully prepared by DC arc plasma gas evaporation and used to make LEDs. The EL emission of LEDs was measured to evaluate the p-type features of nitrogen-doped ZnO NPs, and the EL intensities were found to be proportional to the nitrogen concentrations in the ZnO NPs. The overall experimental analysis showed nitrogen dopant was likely acting as an acceptor for ZnO NPs. These results implied that the fabrication of high-performance ZnO NP-based LEDs could be expected by optimizing the nitrogen concentration in the ZnO NPs.

## Data Availability

The data that support the finding of this study are available on request from the corresponding author. The data are not publicly available due to privacy or ethical restrictions.
